# Dispersion Compensation and Multi-Beam Interference Correction Algorithm for Thickness Measurement of SiC Epitaxial Layer

**DOI:** 10.3390/s26102965

**Published:** 2026-05-08

**Authors:** Lu Liu, Weiwei Shi, Shibo Xu, Xiaofan Wang

**Affiliations:** 1 School of Computer Science and Engineering, Xi’an University of Technology, Xi’an 710048, China; 3230911007@stu.xaut.edu.cn (L.L.); wangxfok@xaut.edu.cn (X.W.); 2 Shaanxi Key Laboratory of Network Computing and Security Technology, Xi’an University of Technology, Xi’an 710048, China; 3 International Engineering College, Xi’an University of Technology, Xi’an 710054, China; 3242231059@stu.xaut.edu.cn

**Keywords:** SiC epitaxial layer, thickness estimation, infrared reflectance spectra, dispersion compensation, multi-beam interference correction, Monte Carlo analysis

## Abstract

**Highlights:**

**What are the main findings?**

- The core bottlenecks of traditional infrared interferometry for thickness measurement are revealed, including dispersion effect, multi-beam interference (interface reflectivity ≥ 17%), and noise superposition.
- A multi-algorithm collaborative scheme of “Savitzky–Golay filtering + Gaussian fitting + Sellmeier equation + Airy function adaptive filtering” is proposed, reducing the inter-angle consistency deviation of SiC epitaxial layers from 1.14% to 0.08%.
- A modular five-layer measurement architecture is constructed to achieve deep integration of physical modeling and computer algorithms. Verified by Monte Carlo simulation, the measurement results maintain high reliability under ±0.1% peak disturbance and Gaussian noise.

**What are the implications of the main findings?**

- It provides a physics-constrained framework for reflectance spectrum-based thickness estimation in SiC-related measurement scenarios and supports more robust analysis under dispersion and multi-beam interference conditions.
- It illustrates how physically grounded modeling and computational signal processing can be integrated in optical thickness evaluation problems, with potential extensibility to additional reflectance spectrum-based applications.

**Abstract:**

To address the main challenges in thickness estimation of SiC epitaxial layers from infrared reflectance spectra, including refractive index dispersion, multi-beam interference, and spectral uncertainty, this study develops a physics-constrained inversion framework for reflectance spectrum-based analysis. For the measured spectra, Savitzky–Golay filtering is first used to suppress spectral noise, and Gaussian fitting is then employed to improve the localization of interference extrema. The Sellmeier equation is introduced to characterize refractive index dispersion, and the layer thickness is obtained together with the dispersion parameters through nonlinear least squares fitting. To account for spectra affected by higher-order internal reflections, a multi-feature confidence-based identification strategy is further constructed, and an adaptive filtering procedure is introduced for multi-beam interference correction. A Monte Carlo perturbation analysis with ±0.1% peak perturbations and Gaussian noise is additionally performed to assess the robustness of the inversion results. Using SiC datasets measured at two incident angles, the proposed framework reduces the inter-angle deviation of the thickness estimates from 1.14% to 0.08% after multi-beam correction. The results support the effectiveness and robustness of the proposed workflow for the main SiC application scenario considered in this study. In addition, silicon wafer spectra are included as a supplementary transfer test to examine whether the multi-beam identification and correction strategy can be applied beyond the SiC example, rather than as a comprehensive cross-material validation of the framework.

## 1. Introduction

The global semiconductor industry is undergoing a profound transformation driven by third-generation semiconductor materials, with silicon carbide (SiC) standing out as a key material due to its unparalleled performance advantages [1,2]. Endowed with high breakdown electric field, excellent thermal conductivity, and exceptional high-temperature stability, SiC has become irreplaceable in critical fields such as new energy vehicle power devices, aerospace electronic systems, and 5G communication infrastructure [1,3]. Epitaxial layer thickness is an important parameter affecting the electrical performance and long-term reliability of SiC-based devices, and reliable thickness evaluation is therefore relevant to process control and quality assessment. As semiconductor manufacturing continues toward higher precision and miniaturization, accurate and non-destructive thickness evaluation methods have become increasingly important in practical engineering applications.

**Figure 1:**
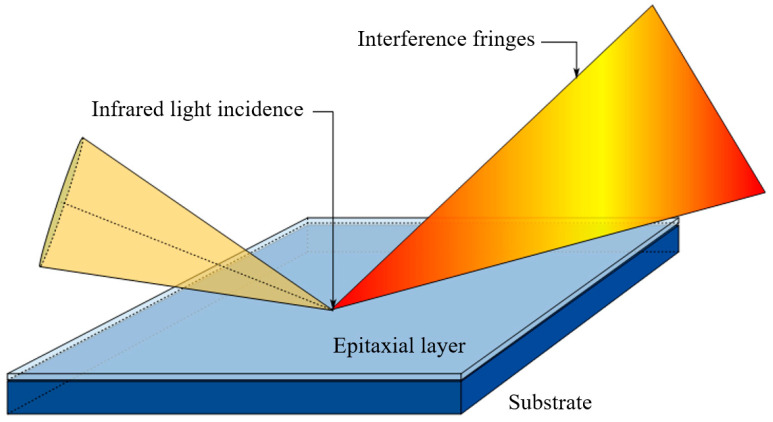
Schematic diagram of infrared interferometry measurement.

Infrared interferometry has been widely adopted in industrial mass production due to its non-contact operation, high efficiency, and broad measurement range [4]. This technique relies on the coherent superposition of reflected light from the epitaxial layer structure, with the basic physical process illustrated in Figure 1.

However, conventional dual-beam interference models face inherent limitations in practical applications, primarily attributed to dispersion effects, environmental noise interference, and multi-beam interference superposition [5]. Existing research efforts often fail to fully address these complex physical phenomena or lack systematic correction methodologies and comprehensive uncertainty quantification [6,7]. For example, Fralaide et al. employed nano-infrared imaging to investigate doping and thickness inhomogeneities of epitaxial graphene on SiC but did not account for measurement deviations induced by multi-beam interference [3]. Afanasjev et al. proposed a frequency analysis approach for infrared reflection spectra to determine the thickness and doping characteristics of multilayer 4H-SiC structures, yet overlooked the wavelength-dependent refractive index variations caused by dispersion [4]. Yoshikawa et al. utilized Fourier transform infrared spectroscopy for characterizing thermal oxides on 4H-SiC epitaxial substrates and reported agreement with ellipsometry within ±1 nm [7]. While this level of performance is suitable for ex situ laboratory characterization under idealized assumptions, such as known optical constants, abrupt interfaces, and single-beam interference, it lacks formal uncertainty quantification and may not meet the stringent demands of in-line process monitoring in advanced semiconductor manufacturing, where sub-nanometer precision, real-time capability, and traceable measurement uncertainty are increasingly required.

To overcome these challenges, this study integrates state-of-the-art computational algorithms with fundamental optical principles, drawing on advanced techniques from related research [8,9,10,11,12,13,14,15,16,17,18,19]. Specifically, Monte Carlo methods are employed for uncertainty quantification [8,9,10,11], optimized filtering techniques for noise suppression [12,19], Airy function-based modeling for interference description [13,14,15,16], multi-beam interference generation mechanisms for interference analysis [17], and adaptive correction strategies for error mitigation [18]. A comprehensive physics-constrained inversion framework is thereby proposed, centered on a five-layer computer-aided measurement architecture. This architecture incorporates Savitzky–Golay filtering to suppress spectral noise, nonlinear least squares fitting to compensate for dispersion effects, and multi-feature confidence assessment to identify multi-beam interference. By systematically addressing noise interference, dispersion effects, and multi-beam interference, this study achieves a significant reduction in the inter-angle consistency deviation of SiC epitaxial layer thickness estimates from 1.14% to 0.08%. Furthermore, the proposed method demonstrates a supplementary transfer test on silicon wafer spectra by successfully identifying multi-beam interference regions and accurately calculating a thickness of 3.442 μm for silicon wafers. This work provides a physics-constrained reflectance spectrum inversion workflow for the SiC thickness estimation scenario considered here, with explicit treatment of dispersion, multi-beam interference, and spectral uncertainty. Its methodological design is broadly consistent with prior studies that combine optical modeling, sensing, and computational analysis in measurement-related applications.

In practical measurements, affected by factors such as dispersion effects, noise interference, and multi-beam interference superposition, the traditional dual-beam interference model suffers from insufficient accuracy. Many existing studies treat some of these effects individually, but systematic integration of correction strategies and uncertainty-aware analysis remains limited for the present SiC application scenario. In optical parameter inversion for thin films and epitaxial layers, commonly used refractive index representations include the constant-index approximation, Cauchy-type empirical dispersion relations, Sellmeier-type parameterizations, and more complex material- or spectral-range-specific models [20,21,22,23]. The suitability of these models generally depends on the material system, the spectral range of interest, the identifiability of the parameters, and the type of available observables. For the present SiC thickness inversion problem based on infrared reflectance spectra, this study does not attempt to perform a systematic model selection study among all possible dispersion forms. Instead, the Sellmeier form is adopted here as a wavelength-dependent refractive index parameterization with relatively moderate model complexity and clear physical interpretability, while remaining convenient for integration into the present joint-fitting workflow. The focus of this work is therefore on constructing and evaluating a physics-constrained inversion workflow for the present SiC scenario, rather than on establishing a universally optimal dispersion model for all materials and spectral ranges. Motivated by these issues, this study develops a physics-constrained inversion workflow that combines dispersion-aware fitting with multi-beam identification and correction, aiming to improve the internal consistency and robustness of SiC epitaxial layer thickness estimation. The SiC epitaxial layer thickness measurement system designed in this study adheres to the core philosophy of “digitalization of physical modeling, algorithmization of data processing, and intellectualization of error correction” and constructs a five-layer computer-aided measurement architecture consisting of “data input—preprocessing—model solving—error correction—result output”. More broadly, recent interferometric strategies, such as polarization phase-shifting Sagnac interferometry for solid films [24], coherence scanning interferometry for buried interfaces [25], and fringe-based thickness tracking in fluid films [26], have achieved high precision under controlled conditions. However, their reliance on simplified optical assumptions (e.g., known dispersion, negligible higher-order reflections, or idealized interfaces) may limit applicability in real-world semiconductor or coating processes, where material variability and noise are significant. Although the proposed framework is formulated in a generally applicable reflectance spectrum inversion form, the present study is primarily developed and validated for SiC epitaxial layer thickness estimation, which is the main application scenario considered in this work. The silicon wafer case included later is intended only as a supplementary transfer test for the correction strategy, rather than a comprehensive validation of general applicability. The contribution of this work does not lie in introducing entirely new individual optical or signal-processing components, since Savitzky–Golay filtering, Gaussian peak fitting, Sellmeier dispersion modeling, Airy-function-based interference analysis, and Monte Carlo perturbation methods are all established techniques. Instead, the contribution lies in integrating these components into a physics-constrained reflectance spectrum inversion workflow tailored to the SiC thickness estimation scenario considered here, with explicit treatment of dispersion, multi-beam interference, and uncertainty-aware evaluation.

It should be noted that the publicly available CUMCM 2025 dataset used in this study does not provide independently calibrated absolute thickness labels such as SEM, ellipsometry, or certified reference values. Therefore, the present study is not intended as a direct metrological accuracy validation. Instead, the evaluation is framed under limited reference conditions. Specifically, the official reference intervals associated with the dataset, namely 7.5–8.5 μm for the SiC sample and 3–4 μm for the silicon wafer sample, are treated only as coarse external plausibility references, while the agreement between independently measured spectra at 10° and 15° incidence is used as the primary internal consistency criterion. On this basis, additional component isolation comparisons and uncertainty analysis are introduced to assess the respective roles of dispersion-aware inversion, multi-beam correction, and robustness under spectral perturbations.

The primary contributions of this paper are listed as follows:

- A physics-constrained inversion framework is established for SiC epitaxial layer thickness estimation from infrared reflectance spectra, with dispersion-aware fitting and multi-beam correction integrated into a unified workflow.
- An adaptive multi-beam correction strategy is introduced to improve the cross-angle consistency of thickness estimates on the SiC datasets, as demonstrated through comparative analysis before and after correction.
- A supplementary transfer test on silicon wafer spectra is conducted to examine the applicability of the multi-beam identification and correction strategy beyond the main SiC application scenario.

## 2. Materials and Methods

### 2.1. Basic Theory

#### 2.1.1. Digital Modeling of Dual-Beam Interference

For the present reflectance-based thickness estimation problem, the epitaxial layer system is described within a thin-film interference framework. The amplitude reflection coefficients at the air–epitaxial layer interface and the epitaxial layer–substrate interface are given by [27]:

(1) $$r_{01}=\frac{n_{0}-n_{1}}{n_{0}+n_{1}}$$

(2) $$r_{12}=\frac{n_{1}-n_{2}}{n_{1}+n_{2}}$$

where $r_{01}$ and $r_{12}$ denote amplitude reflection coefficients at the air–epitaxial layer interface and the epitaxial layer–substrate interface, respectively; $n_{0}$, $n_{1}$, and $n_{2}$ denote the refractive indices of air, the epitaxial layer, and the substrate, respectively. The reflectivity is defined as $R=|r{|}^{2}$.

**Figure 2:**
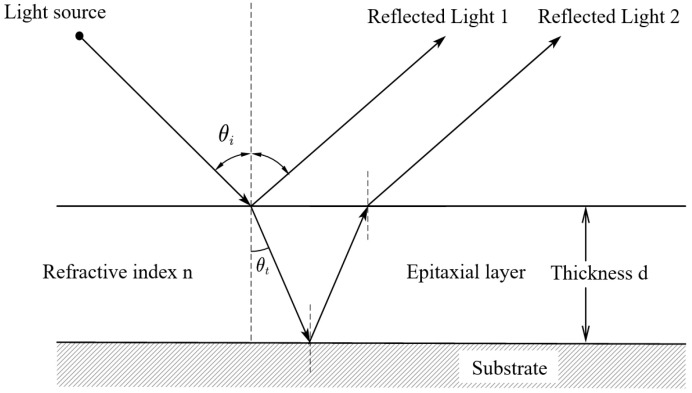
Schematic diagram of two-dimensional geometric relationship in infrared interferometry.

Figure 2 illustrates the geometric relation adopted in the dual-beam approximation. Based on this geometry, the optical path difference associated with the round-trip propagation inside the epitaxial layer is expressed as [27]:

(3) $$\Delta L=2n_{1}dcos\theta_{t}$$

where $\theta_{t}$ is the refraction angle in the film and d denotes the thickness of the epitaxial layer. The corresponding phase difference is written as [27]:

(4) $$\delta =\frac{4\pi n_{1}dcos\theta_{t}}{\lambda }+\Delta \delta$$

where λ is the wavelength and Δδ denotes the additional phase contribution introduced by interfacial phase jumps. Both reflections at the air–epitaxial layer interface and the epitaxial layer–substrate interface generate a phase jump of π, which cancel each other out, leading to Δδ=0.

Accordingly, the spectral extrema can be related to the unknown thickness through the interference condition. For interference minima, the thickness satisfies [27]:

(5) $$d=\frac{\left(2k+1\right)\lambda }{4n_{1}cos\theta_{t}}$$

where k is the interference order. In this study, this relation provides the physical basis for the subsequent thickness inversion, while the practical fitting procedure is introduced in the following sections.

Because the refractive index of SiC varies with wavelength, a constant-index approximation would introduce systematic bias. The wavelength dependence of the refractive index is therefore described using the Sellmeier equation [28]. In the present study, the Sellmeier form is adopted as a wavelength-dependent refractive index parameterization with relatively moderate model complexity and convenient integration into the subsequent joint fitting of thickness and dispersion parameters. It should also be noted that this work does not attempt to perform a systematic model selection study among the Sellmeier, Cauchy, and other empirical or semi-empirical dispersion forms. Therefore, the present choice should not be interpreted as a claim that alternative models are universally inapplicable, but rather as a modeling decision adopted for the current SiC infrared reflectance spectrum inversion scenario. The Sellmeier equation is given by

(6) $$n\left(\lambda \right)=\sqrt{A+\frac{B\lambda^{2}}{\lambda^{2}-C}}$$

where A, B, and C are dispersion parameters. The thickness and dispersion parameters are then determined jointly in the subsequent inversion procedure. Therefore, the above relations constitute the physical basis for the dispersion-aware thickness estimation framework adopted in this study.

#### 2.1.2. Identification and Correction Model for Multiple-Beam Interference

When the reflectivity R at the epitaxial layer–substrate interface is greater than or equal to 17%, coherent superposition of multiple reflected beams (multiple-beam interference) will occur, leading to the sharpening of interference peaks and thus degradation of the solution accuracy of the dual-beam model. The system implements interference processing through the following two steps:

(1) Digital Identification of Multiple-Beam Interference

An intensity distribution model for multiple-beam interference is established based on the Airy function [24].

(7) $$R=\frac{{\left(1-R_{1}R_{2}\right)}^{2}+4R_{1}R_{2}{sin}^{2}\varphi }{{\left(1+R_{1}R_{2}\right)}^{2}+4R_{1}R_{2}{sin}^{2}\varphi }$$

where $R_{1}=|r_{01}{|}^{2}$ and $R_{2}=|r_{12}{|}^{2}$ are the interface reflectivity and $\varphi =\frac{2\pi n_{1}dcos\theta_{t}}{\lambda }$ is the phase difference between adjacent reflected beams. To determine whether multiple-beam interference is present, five quantitative features, including fringe contrast, fringe sharpness, Fourier spectrum quality, periodicity score, and modulation depth, are extracted from the reflectance spectrum and combined using a weighted confidence criterion.

(2) Correction Algorithm for Multiple-Beam Interference

For spectra identified as containing multiple-beam interference, an adaptive filtering method is introduced to suppress the sharp fringe components while retaining the smooth characteristics of dual-beam interference. The correction procedure includes three stages:

(i) Physics-guided feature initialization: key prior information, including fringe period, estimated thickness, and effective refractive index, is extracted from a reference dual-beam spectrum to provide physical constraints for the filtering process.
(ii) Physics-constrained adaptive optimization: the filter parameters are adaptively determined by optimization, with the objective function penalizing deviations from the target fringe period to maintain physical consistency.
(iii) Adaptive filtering execution: the optimized filter is applied in the frequency domain to suppress the multiple-beam oscillation components, followed by a smoothing step to improve signal continuity.

**Figure 3:**
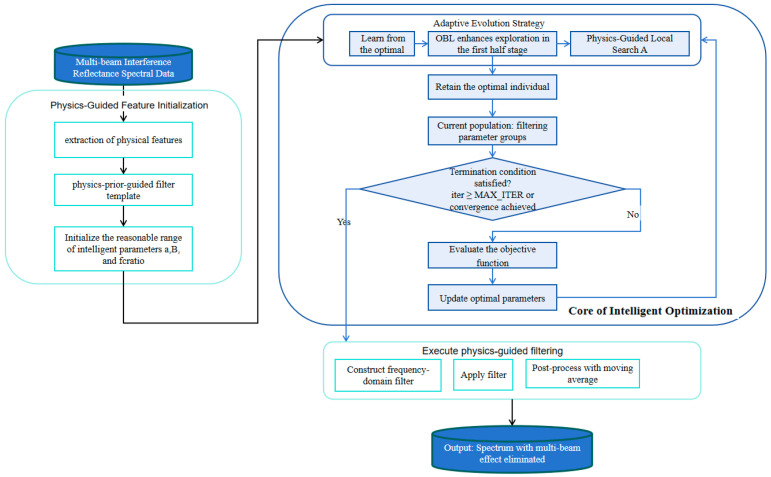
Adaptive filtering and parametric-solving for multi-beam spectra.

After correction, the processed spectrum is reintroduced into the nonlinear joint fitting model for thickness estimation. The overall procedure is illustrated in Figure 3.

### 2.2. Dual-Beam-Based Thickness Inversion for SiC Spectra

#### 2.2.1. Savitzky–Golay Filtering

Raw spectral data is susceptible to random noise induced by measurement environmental factors (e.g., light fluctuation) and instrumental precision limitations, which leads to abrupt values in the subsequently derived reflectivity curves. In this study, the experimental data is processed using a Savitzky–Golay filter, which eliminates high-frequency noise while preserving the peak characteristics of interference peaks. This treatment smooths the reflectivity curves, facilitating the accurate identification of true interference peaks.

Nonsensical negative values may emerge after the smoothing process. To comply with the physical law that “reflectivity ≥0” and avoid errors in subsequent refractive index calculations, the lower bound of the smoothed reflectivity is set to 0 in this study.

#### 2.2.2. Data Processing

(1) Refractive Index Inversion
  Based on the Fresnel formulas, the refractive index is inverted using the reflectivity data.
  (i) Background Reflectivity Fraction
    A wavelength region accounting for the largest 20% of the spectral range is selected (the thin-film interference effect weakens with increasing wavelength, and the reflectivity is solely determined by the air–epitaxial layer interface). The background reflectivity $R_{frac}$ is calculated as follows [27]:
    (8) $$R_{frac}=\frac{background\;reflectance\;(\%)}{100}$$
  (ii) Calculation of Epitaxial Layer Refractive Index
    (9) $$n_{1}=\frac{1+\sqrt{R_{frac}}}{1-\sqrt{R_{frac}}}$$
  (iii) Refractive Index Constraint
    (10) $$n_{1}=clip(n_{1},1.0,3.5)$$
(2) Subpixel Peak Position Fitting

To obtain more accurate wavenumber values of the peaks, a Gaussian function is employed to fit the data points around each peak, with the expression given by [29]:

(11) $$y=a\times \exp\left(-\frac{(x-\mu {)}^{2}}{2\sigma^{2}}\right)+c$$

where a denotes the peak amplitude, μ is the peak position, *σ* is the Gaussian peak width parameter, and c represents the baseline reflectivity. The data used for Gaussian fitting are extracted from the experimental interference reflectance spectra of the thin-film samples. Specifically, the raw reflectance data points around each interference peak are selected as the input for the subpixel peak position fitting. For each interference peak, Gaussian fitting was performed within a local window centered on the peak maximum, using 5 neighboring data points on each side (11 points in total under normal conditions).

(3) Interference Peak Identification
  (i) Local Maximum Judgment: The smoothed reflectivity data is traversed, and points satisfying “current value > values of adjacent left and right points” are identified as potential peaks.
  (ii) Threshold Screening: Only peaks with “reflectivity > mean value + 0.5 × standard deviation” are retained to eliminate false peaks caused by noise.
  (iii) Sorting and Collation: The peaks are sorted in descending order of wavelength, corresponding to interference orders from high to low, which lays the foundation for subsequent linear fitting.

#### 2.2.3. Model Establishment

Traditional simple linear models exhibit inherent limitations because they neglect the wavelength dependence of the refractive index, simplify the role of incident angle, and do not quantify the influence of algorithmic uncertainty on the retrieved results. To address these issues, this study introduces the Sellmeier dispersion model and Monte Carlo perturbation analysis and constructs a thickness inversion method combining nonlinear joint fitting with uncertainty evaluation.

It should also be noted that the present inversion framework is formulated under an effective homogeneous-layer assumption. In other words, the epitaxial layer is treated as an optically effective layer whose behavior is described through an effective refractive index parameterization. Under this assumption, surface crystallinity, interface roughness, thickness/refractive index non-uniformity, and possible polycrystalline microstructural effects are not introduced as independently resolved variables in the current model. Therefore, the present method is not intended as a microstructure-resolved model for general polycrystalline films, but rather as an effective optical inversion framework for the current SiC reflectance spectrum scenario.

(1) Thin-Film Interference Condition

The interference enhancement condition is satisfied when light is incident on the thin-film material.

(2) Derivation of Refraction Angle

According to the law of refraction: $\sin\theta_{i}(\lambda )=\frac{\sin\theta_{1}}{n_{1}(\lambda )}$, where $\theta_{1}$ is the experimentally set incident angle. The cosine of the refraction angle is further calculated as:

(12) $$\cos\theta_{i}(\lambda )=\sqrt{1-\sin^{2}\theta_{i}(\lambda )}$$

(3) Dispersion Model (Sellmeier Equation)

The Sellmeier equation is introduced to characterize the dispersion property of the refractive index varying with wavelength, which enables wavelength-dependent modeling of the refractive index and resolves the assumption bias of a constant refractive index in the unoptimized model.

#### 2.2.4. Nonlinear Fitting Solution Model

(1) Initial Value Estimation
  (i) Initial Thickness Value: The slope m is obtained by linearly fitting the relationship between 1/λ and 2k+1. Combined with the average refractive index $n_{avg}$ inverted from the background reflectivity, the initial thickness value is estimated using the formula [30]:
    (13) $$d_{initial}=\frac{1}{4n_{avg}\cdot m\cdot \cos\theta_{t}}$$
  If the slope m of linear fitting is less than or equal to 0, an empirical value of $d_{initial}=5\;\mu m$ is adopted to avoid abnormal initial values.
  (ii) Initial Values of Sellmeier Parameters: Referring to the empirical dispersion parameters of SiC thin-film materials, the initial values are set as A=6.45, B=2.14, and $C=0.65\;{\mu m}^{2}$, ensuring that the initial values fall within a physically reasonable range. Parameters A and B are dimensionless, whereas C has the same unit as $\lambda^{2}$. It should be emphasized that these values of A, B, and C are used only as initial guesses for the fitting procedure, rather than as known fixed inputs. In the subsequent inversion, the thickness d and the Sellmeier parameters A, B, and C are treated as jointly unknown parameters and are determined simultaneously through nonlinear fitting.
(2) Objective Function for Nonlinear Joint Fitting

The residual formula essentially represents the sum of squared residuals between the theoretical and measured interference orders, which is directly correlated with the physical model of dual-beam interference and serves as the core quantitative index for evaluating the thickness fitting accuracy. A smaller residual value indicates that the fitting parameters are more consistent with the optical interference laws, and the thickness results are more credible.

Treating the thickness d and the Sellmeier parameters A, B, and C as jointly solved parameters, the objective function is constructed to minimize the residual between the theoretical and measured interference orders:

(14) $$residual=k_{theory}-k_{practical}$$

where $k_{theory}=\frac{2n_{1}(\lambda )d\;\cos\theta_{i}(\lambda )}{\lambda }$ denotes the theoretical interference order and $k_{practical}$ is the actual interference order extracted from the spectral data. The nonlinear problem is solved via the least squares method, with d, A, B, and C being obtained simultaneously.

### 2.3. Advanced Modeling with Multiple-Beam Interference Consideration

#### 2.3.1. Fundamental Criteria

Multiple-beam interference originates from the successive reflections of light at the epitaxial layer–substrate interface, which requires two essential prerequisites (fundamental criteria):

High Interface Reflectivity Criterion: The reflectivity at the epitaxial layer–substrate interface must be sufficiently high (typically ≥ 17%), ensuring that the intensity of subsequent reflected beams attenuates slowly and can participate in superposition to form valid interference. This threshold originates from Fabry-Pérot etalon theory: when the interface reflectivity R satisfies R⪆0.17, the finesse $F=\frac{\pi \sqrt{R}}{1-R}$ exceeds unity (F>1), indicating that higher-order reflections significantly contribute to the interference pattern and the dual-beam approximation becomes inadequate [27].

For silicon as the test object, the epitaxial layer–substrate interface inherently exhibits a high reflectivity, which satisfies this basic criterion.

Stable Phase Difference Criterion: The optical path difference between adjacent reflected beams must remain stable, which is jointly determined by the epitaxial layer thickness, refractive index, and the incident angle of light. This criterion is reflected in the spectral data as stable periodicity of interference fringes.

#### 2.3.2. Data Preprocessing

The characteristic features of multiple-beam interference fringes (e.g., sharpness and periodicity) are vulnerable to measurement noise; thus, the raw reflectivity data must be subjected to smoothing and denoising first. The Savitzky–Golay filtering method is adopted, which not only preserves fringe features but also filters out high-frequency noise, providing a reliable data foundation for subsequent feature extraction.

#### 2.3.3. Key Feature Extraction

Multiple-beam interference in reflection spectra typically exhibits several distinguishable spectral characteristics, including relatively sharp fringes, high contrast, and stable periodicity. Consistent with previous studies on interference-based spectral analysis and signal quality assessment (e.g., Afanasjev et al. [4] and Yoshikawa et al. [7]), five physically motivated quantitative features are extracted from the smoothed data to characterize complementary aspects of the interference pattern:

(1) Fringe Contrast

The peak and valley values of the reflectivity curve are first identified. To reduce the influence of abnormal extrema caused by spectral noise, outliers are excluded by retaining only the data within the 25th–75th percentile range. The average peak reflectivity ${\bar{R}}_{max}$ and average valley reflectivity ${\bar{R}}_{min}$ are calculated. The contrast formula is given by [31]:

(15) $$C=\frac{{\bar{R}}_{max}-{\bar{R}}_{min}}{{\bar{R}}_{max}+{\bar{R}}_{min}}$$

In general, spectra exhibiting clearer multiple-beam interference characteristics tend to show higher fringe contrast than spectra dominated by dual-beam interference or spectral noise.

(2) Fringe Sharpness

Multiple-beam interference fringes usually exhibit relatively steep edges and well-defined extrema, which can be characterized by fringe sharpness [27]. The sharpness parameter is defined as

(16) $$S=\frac{\Delta \lambda_{FSR}}{\Delta \lambda_{FWHM}}$$

where $\Delta \lambda_{FSR}$ refers to the free spectral range (wavelength spacing between adjacent fringes) and $\Delta \lambda_{FSR}={\bar{R}}_{max}-{\bar{R}}_{min}$. $\Delta \lambda_{FWHM}$ refers to the full width at half maximum (FWHM) of a single fringe.

(3) Fourier Spectrum Quality

Multiple-beam interference spectra generally exhibit relatively stable fringe periodicity, which corresponds to a pronounced dominant peak and comparatively weak background interference in the Fourier spectrum. To characterize this feature, the Fourier spectrum quality is defined as [32]:

(17) $$Q=\frac{I_{main}}{\Sigma_{K=1}^{N}I_{harm,k}}$$

where $I_{main}$ refers to the intensity of the main peak in the Fourier spectrum and $\Sigma_{K=1}^{N}I_{harm,k}$ refers to the sum of intensities of all harmonic peaks. A higher Q indicates a clearer dominant periodic component in the spectrum.

(4) Periodicity Score

Regular fringe spacing is an important observable characteristic of multiple-beam interference. To quantify the uniformity of fringe spacing, the periodicity score is defined as [33]:

(18) $$P=\frac{1}{1+\frac{\sigma (\Delta \lambda )}{\mu (\Delta \lambda )}}$$

where μ(Δλ) refers to the mean value of the wavelength spacing between adjacent fringes and σ(Δλ) refers to the standard deviation of the wavelength spacing between adjacent fringes.

(5) Modulation Depth

Multiple-beam interference spectra often exhibit relatively large peak-to-valley variations. The modulation depth is therefore defined as

(19) $$M=\frac{I_{max}-I_{min}}{I_{max}}$$

where $I_{max}$ refers to the maximum intensity of the interference fringes and $I_{min}$ refers to the minimum intensity of the interference fringes.

#### 2.3.4. Comprehensive Identification of Multiple-Beam Interference

A combined strategy of weighted scoring and feature verification is adopted to avoid misjudgment caused by a single feature, ensuring the reliability of the identification results.

Step 1: Calculation of Comprehensive Confidence

Different weights are assigned to the five features as follows: contrast (0.25), sharpness (0.20), Fourier spectrum quality (0.25), periodicity (0.15), and modulation depth (0.15). Each feature value is first normalized to a score ranging from 0 to 1, followed by the calculation of a weighted total score. A comprehensive confidence score closer to 1 indicates a higher probability of the presence of multiple-beam interference.

Step 2: Result Determination via Rule Verification

The presence of multiple-beam interference is determined if any of the following rules is satisfied: comprehensive confidence > 0.70 (direct determination with high confidence); confidence > 0.65, with Fourier spectrum quality Q≥ 6 and fringe sharpness S ≥2.5; confidence > 0.60, with fringe contrast C ≥ 0.2 and periodicity score P ≥ 8.5.

#### 2.3.5. Correction with the Introduction of Airy Function

Data preprocessing has been completed in the preceding sections (including wavelength conversion, Savitzky–Golay filtering, and the Sellmeier equation application). All subsequent modeling and validation steps are implemented based on this cleaned dataset. Wavelength conversion is the reciprocal of wavelength, which satisfies the following relationship:

(20) $$\lambda =\frac{1}{\nu }$$

(1) Calculation of Fresnel Reflectivity

To make the calculation procedure more applicable to practical thickness estimation scenarios, the Fresnel reflectivity expression for normal incidence is adapted for the present modeling framework. To avoid interference from the reflectivity variation of p-polarized light near the Brewster angle, calculations are only performed for s-polarized light. The reflection coefficient of s-polarized light $r_{s}$ is expressed as [27]:

(21) $$r_{s}=\frac{\cos\theta_{1}-n\cos\theta_{2}}{\cos\theta_{1}+n\cos\theta_{2}}$$

where $\theta_{1}$ is the incident angle, $\theta_{2}$ is the refraction angle in silicon, and n is the dispersive refractive index of silicon.

(2) Construction of Interference Model

Based on the dual-beam interference scenario involving single reflection and transmission at the interface, the Airy function is known as: $R=\frac{r_{01}^{2}+2r_{01}r_{12}\cos\delta +r_{12}^{2}}{1+2r_{01}r_{12}\cos\delta +r_{01}^{2}r_{12}^{2}}$, where δ=4πndcosθ. The relationship between the theoretical reflectivity and thickness is established using the Airy function, with the expression given by [27]:

(22) $$R(d)=\frac{R_{0}}{1+\frac{4R_{0}}{(1-R_{0}{)}^{2}}\cdot \sin^{2}\left(\frac{2\pi nd\cos\theta_{t}}{\lambda }\right)}$$

(3) Initial Thickness Estimation

To prevent the subsequent optimization algorithm from falling into a local optimum, the initial thickness is estimated first using the characteristics of interference fringes.

(4) Optimization Solution

Residual minimization is applied to obtain the true thickness value d. The optimization objective is defined as minimizing the sum of squared residuals between the measured and theoretical reflectivity, with the objective function expressed as [34]:

(23) $$J(d)=\sum_{i=1}^{N}(R_{practical}(\lambda_{i})-R_{theory}(d,\lambda_{i}){)}^{2}$$

where $J\left(d\right)$ is the objective function, the sum of squared residuals between measured and theoretical reflectivity. Iterative optimization is simultaneously implemented using the fminbnd function (an interval optimization algorithm). The thickness value that minimizes the objective function is iteratively searched within the reasonable interval of 0.5 d to 2 d to obtain the most accurate result.

## 3. Results

### 3.1. Dual-Beam Interference-Based Solution

#### 3.1.1. Calculation Results of Epitaxial Layer Thickness

**Figure 4:**
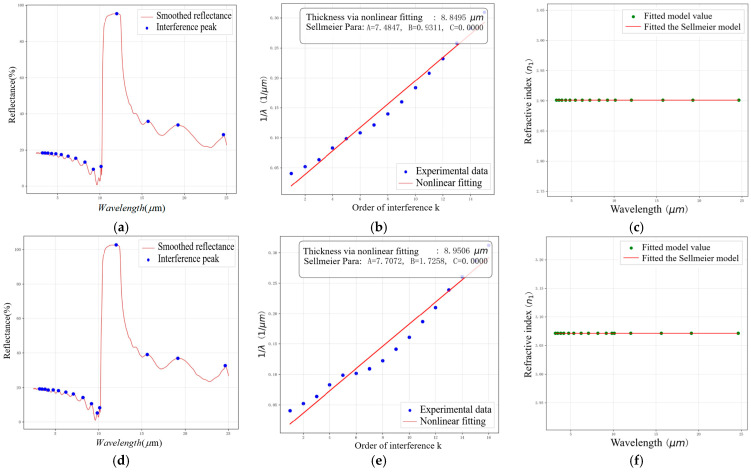
Schematic diagram of reflection spectra and fitting results at 10° and 15° after model optimization: (**a**) reflectance spectrum at incidence = 10°; (**b**) interference order and wavelength at incidence = 10°; (**c**) dispersion curve at incidence = 10°; (**d**) reflectance spectrum at incidence = 15°; (**e**) interference order and wavelength at incidence = 15°; (**f**) dispersion curve at incidence = 15°.

The calculated thickness results obtained from the nonlinear inversion are presented in Table 1 and Figure 4. In particular, the fitted value $C\approx 10^{-6}$ indicates weak sensitivity of the present dataset to this term within the adopted parameterization, rather than the absence of refractive index dispersion in a general sense. Figure 4 presents the reflection spectra of the SiC epitaxial layer and the corresponding theoretical fitting curves at incident angles of $10^{\circ }$ (Figure 4a) and $15^{\circ }$ (Figure 4b). The experimental data (scatter points) closely align with the fitted curves (solid lines), indicating that the adopted wavelength-dependent refractive index parameterization, combined with nonlinear least squares optimization, can reproduce the main interference characteristics of the measured spectra over the present spectral interval. The sharp interference peaks are well preserved after Savitzky–Golay filtering, and their positions are reasonably matched by the fitted model, supporting effective suppression of spectral noise and reliable extraction of interference orders. Notably, the fitted Sellmeier parameters (A,B,C) in Table 1 exhibit physical consistency, with the retrieved thickness values of 8.8495 μm ($10^{\circ }$) and 8.9506 μm ($15^{\circ }$) showing good agreement between the two angle-based estimates. The remaining deviations suggest that the inversion results may still be influenced by modeling assumptions in the baseline framework as well as by residual multi-beam interference effects, which are further examined in the following sections.

#### 3.1.2. Assessment of the Constant-Index Assumption in Thickness Inversion

To assess the role of refractive index dispersion in the present SiC thickness inversion problem, an additional comparison was performed using a simplified constant-index model. In this comparison, the refractive index was treated as wavelength-independent, and the thickness was determined from the linear relationship between interference order and inverse wavelength, while the peak identification procedure was kept unchanged. This simplified treatment corresponds to a reduced-form dual-beam inversion without explicit dispersion modeling.

**Figure 5:**
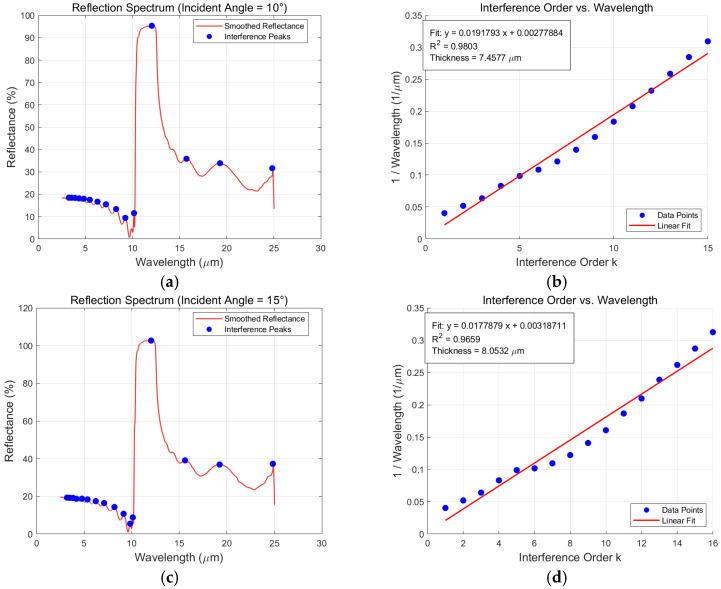
Schematic diagram of reflection spectra and fitting results at 10° and 15° under the simplified constant-index assumption: (**a**) reflectance spectrum at incidence = 10°; (**b**) interference order and wavelength at incidence = 10°; (**c**) reflectance spectrum at incidence = 15°; (**d**) interference order and wavelength at incidence = 15°.

The corresponding results are shown in Figure 5. Under the constant-index assumption, the retrieved thicknesses are 7.4577 μm for the 10° dataset and 8.0532 μm for the 15° dataset. Compared with the dispersion-aware inversion results in Table 1, the discrepancy between the two independently measured angle-based estimates becomes noticeably larger when the wavelength dependence of the refractive index is ignored. This indicates that the constant-index approximation is not sufficient to maintain consistent thickness inversion across different incidence angles for the present SiC spectra.

This difference can be attributed to the fact that SiC exhibits non-negligible refractive index dispersion over the analyzed spectral range. When this wavelength dependence is neglected, the interference–order–wavelength relation is represented with reduced physical fidelity, which introduces accumulated bias into the linear fitting process and consequently reduces the cross-angle consistency of the retrieved thickness values. Therefore, for the present SiC reflectance spectrum inversion problem, dispersion-aware modeling is not merely an auxiliary refinement, but an essential component of the baseline inversion framework.

For clarity, the constant-index comparison in this section serves to isolate the effect of dispersion modeling within the baseline dual-beam framework. The above comparison shows that dispersion modeling is already necessary within the baseline inversion stage, before the additional influence of multi-beam interference is considered.

#### 3.1.3. Uncertainty Analysis via Monte Carlo Simulation

The Monte Carlo method is a computational technique based on random number generation, also referred to as stochastic or statistical computer simulation, which falls within the realm of computational mathematics. Directly fitted single results may lack reliability due to the influence of spectral noise and instrumental errors. Therefore, Monte Carlo simulation was performed on the obtained data, with the detailed steps outlined as follows:

(1) Perturbation Setup

Perturbation was applied to the peak wavelengths using the formula:

(24) $$\lambda_{perturbed}=\lambda_{ord}\cdot (1+N(0,0.001))$$

Gaussian noise was added to the reflectivity data, where the noise level was set as 0.3 times the standard deviation of the first 100 reflectivity data points. Subsequently, peak detection was repeated, with 80–100% of the peaks randomly selected for calculation in each iteration.

(2) Fitting and Statistics

Nonlinear joint fitting was repeated after each perturbation, and the successfully fitted thickness values $d_{mc}$ and Sellmeier parameters were recorded. The iteration was terminated if fitting failed consecutively for 50 times.

(3) Result Analysis

The mean value $d_{mean}$ and standard deviation $d_{std}$ were calculated from the successfully fitted $d_{mc}$. The 95% confidence interval was obtained using the formula:

(25) $$d_{ci}=1.96\times \frac{d_{std}}{\sqrt{N_{success}}}$$

where $d_{Ci}$ refers to the margin of error for the 95% confidence interval, i.e., it represents the allowable deviation between the measured values and the true value under a 95% confidence level. The value 1.96 refers to the Z-score corresponding to the 95% confidence level, i.e., a critical value commonly used in statistics to calculate 95% confidence intervals. $d_{std}$ refers to the standard deviation of the successfully measured data, i.e., it reflects the dispersion of results from repeated successful measurements. $N_{success}$ refers to the number of successful measurements, i.e., the count of valid measurements included in the statistical analysis.

**Figure 6:**
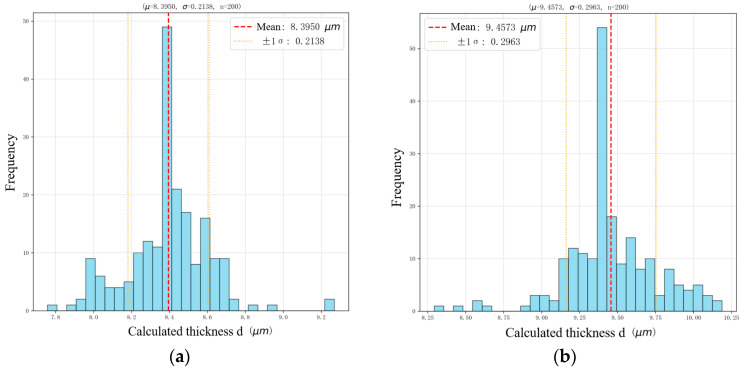
(**a**) Histogram of Monte Carlo simulated thickness at 10°; (**b**) histogram of Monte Carlo simulated thickness at 15°. Data source: Monte Carlo simulation.

Monte Carlo simulation was used to evaluate the robustness of the retrieved thickness under peak perturbations and spectral noise. Figure 6 shows the normal distributions of thickness values from 200 Monte Carlo iterations (with ±0.1% peak perturbation and Gaussian noise). For 10° incidence (Figure 6a), the mean (μ) is 8.3950 μm and standard deviation (σ) is 0.2138 μm; for 15° incidence (Figure 6b), μ = 9.4573 μm and σ = 0.2963 μm. Both distributions are symmetric with no outliers, and the narrow 95% confidence intervals (10°: [8.3651 μm, 8.4249 μm]; 15°: [9.4157 μm, 9.4989 μm]) confirm the robustness and reliability of the measurement model against perturbations.

### 3.2. Multiple-Beam Interference-Based Solution and Extension

#### 3.2.1. Identification for Attachments S1 and S2 (SiC Samples)

The multiple-beam interference identification model established in Section 2 was applied to analyze the data from Attachment S1 and Attachment S2 (see Supplementary Materials), and the confidence levels indicating the presence of multiple-beam interference were calculated. A confidence level exceeding 70% indicates that the experimental data in Attachments S1 and S2 were affected by multiple-beam interference. Since the previous thickness calculation for SiC epitaxial layers only considered dual-beam interference, it is necessary to eliminate the influence of multiple-beam interference on the experimental data.

**Figure 7:**
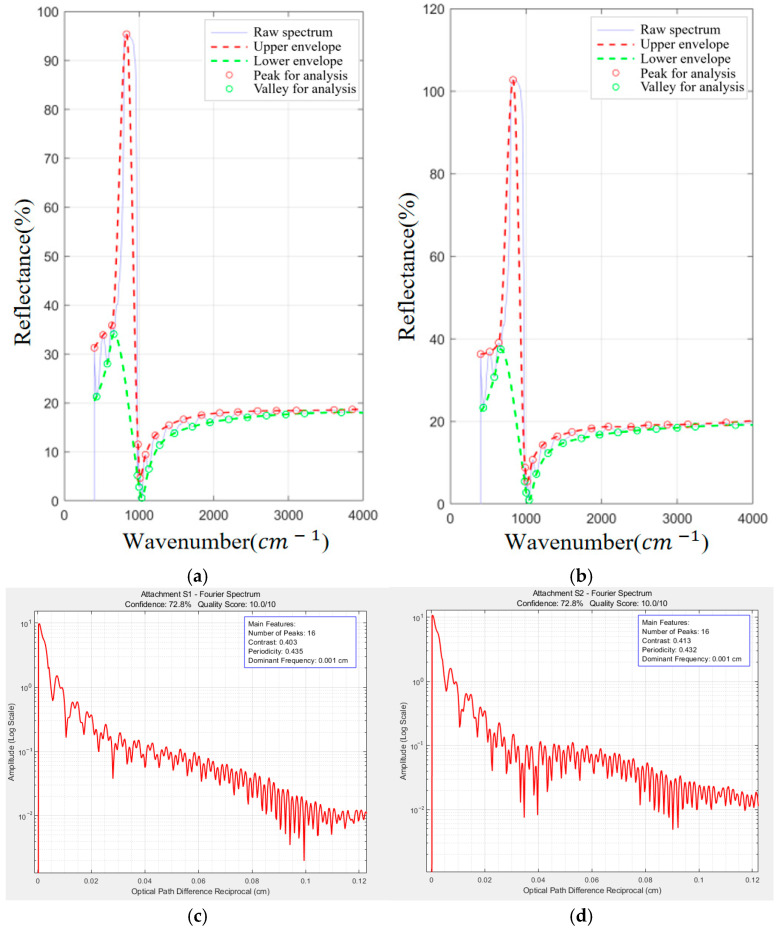
Schematic diagrams of reflection spectra and envelope curves, Fourier spectrum, and radar chart: (**a**) reflectance spectrum and envelope at incidence = 10°; (**b**) reflectance spectrum and envelope at incidence = 15°; (**c**) Fourier spectrum at incidence = 10°; (**d**) Fourier spectrum at incidence = 15°.

**Figure 8:**
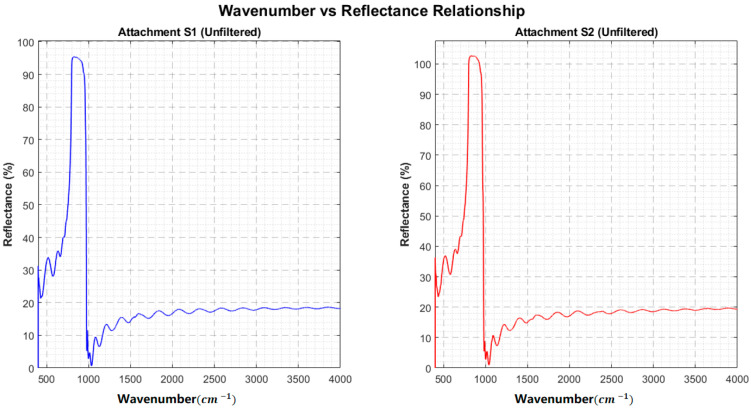
Wavenumber vs. reflectance relationship diagram for Attachments S1 and S2 without filtering.

Figure 7 provides a multi-faceted visualization of the multi-beam interference identification process for SiC samples (Attachments S1 and S2). The upper panels display the original reflection spectra and their Savitzky–Golay smoothed envelopes, highlighting the preservation of fringe structure post-filtering. The Fourier spectrum (middle panel) exhibits a dominant peak with minimal harmonic distortion, confirming strong periodicity, a hallmark of multi-beam interference. The resulting composite confidence level of 72.8% (see Table 2) robustly supports the presence of multi-beam interference in both 10° and 15° configurations, necessitating the application of the adaptive correction algorithm. Figure 8 provides wavenumber vs. reflectance relationship diagrams for Attachments S1 and S2 without filtering.

#### 3.2.2. Adaptive Filtering Correction for SiC Thickness

This study employs an adaptive filtering algorithm to suppress multi-beam interference while retaining dual-beam interference features. After eliminating the multi-beam interference component, the processed data are applied to the dual-beam interference model for thickness optimization. The uncorrected case corresponds to direct inversion of the raw spectra before activating the multi-beam correction branch. The corrected case corresponds to re-inversion after the multi-beam correction step. The corrected and uncorrected comparison results are then compared and analyzed.

Comparison of the results in Table 3 and Table 4 shows that the inter-angle consistency deviation is reduced from 1.14% (uncorrected, Table 4) to 0.08% (corrected via adaptive filtering, Table 3), which verifies the effectiveness of the proposed correction algorithm in mitigating the adverse effects of multi-beam interference. Since the CUMCM 2025 dataset does not include any absolute thickness reference (e.g., from SEM or ellipsometry), model performance is assessed by the agreement between thickness estimates obtained from independent measurements at 10° and 15° incidence angles. Data from Attachments S3 and S4 are used to extract five key quantitative features; their weights and scores are computed to determine the comprehensive confidence level, enabling identification of multi-beam interference.

#### 3.2.3. Identification for Attachments S3 and S4 (Silicon Wafer Samples)

The silicon wafer results presented in this section are intended as a supplementary transfer test beyond the main SiC application scenario. They are used to examine whether the multi-beam identification and correction strategy can be transferred to another reflectance spectrum case, rather than to claim a comprehensive cross-material validation of the entire framework.

The data from Attachments S3 and S4 were substituted into the aforementioned model to extract five key quantitative features. The weights and scores of these features were calculated to obtain the comprehensive confidence levels, thereby determining the presence of multiple-beam interference.

The confidence levels of both Attachments S3 and S4 exceed 70%, indicating a high probability of multiple-beam interference in these samples. Similar multi-feature fusion identification strategies have been applied in the optical detection of material defects, such as blackhearted potatoes.

**Figure 9:**
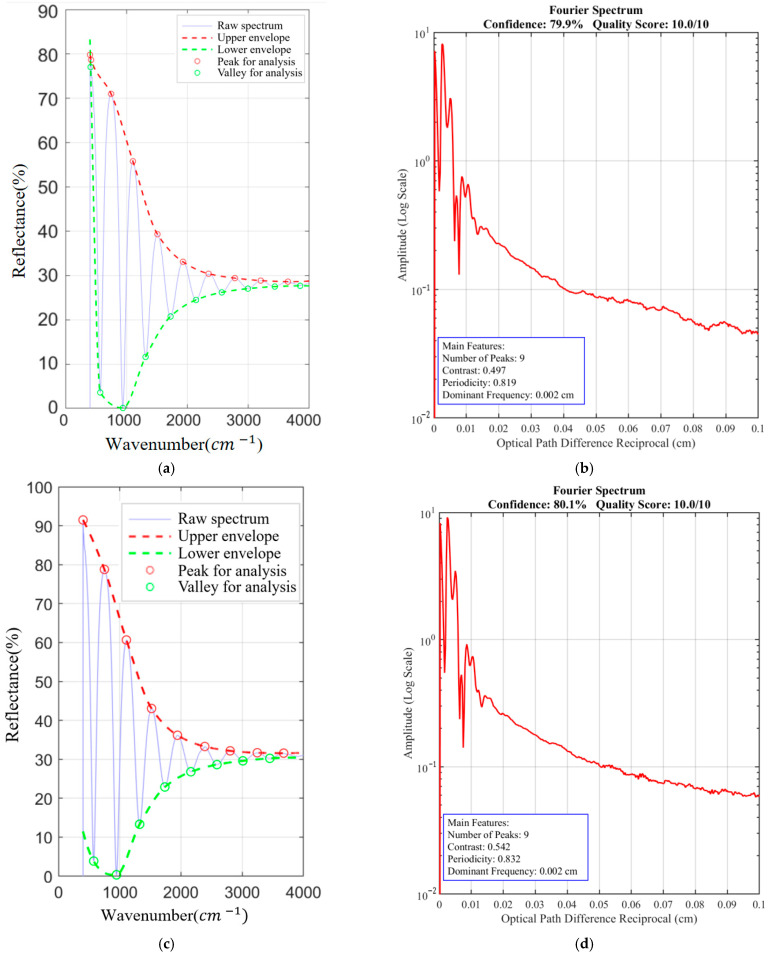
Schematic diagrams of reflection spectra and envelope curves, and Fourier spectrum: (**a**) reflectance spectrum and envelope at incidence = 10°; (**b**) Fourier spectrum at incidence = 10°; (**c**) reflectance spectrum and envelope at incidence = 15°; (**d**) Fourier spectrum at incidence = 15°.

Figure 9 illustrates the reflection spectra, envelope curves, and Fourier spectra for silicon wafer samples (Attachments S3 and S4). The envelope curves exhibit pronounced, high-contrast fringes with steep edges, characteristic of strong multi-beam interference. The corresponding Fourier spectra show sharp primary peaks with high signal-to-noise ratios, further corroborating the stable periodic nature of the interference patterns. These visual indicators align with the high confidence scores of 79.9% and 80.1% reported in Table 5, confirming that silicon wafers are also subject to significant multi-beam interference. This finding suggests that the proposed identification strategy may be applicable beyond the main SiC example in additional reflectance spectrum cases.

#### 3.2.4. Thickness Correction for Silicon Wafers via the Airy Function

For the supplementary silicon wafer case, the Airy-function-based correction improves the consistency between the fitted spectra and the measured reflectance data.

**Figure 10:**
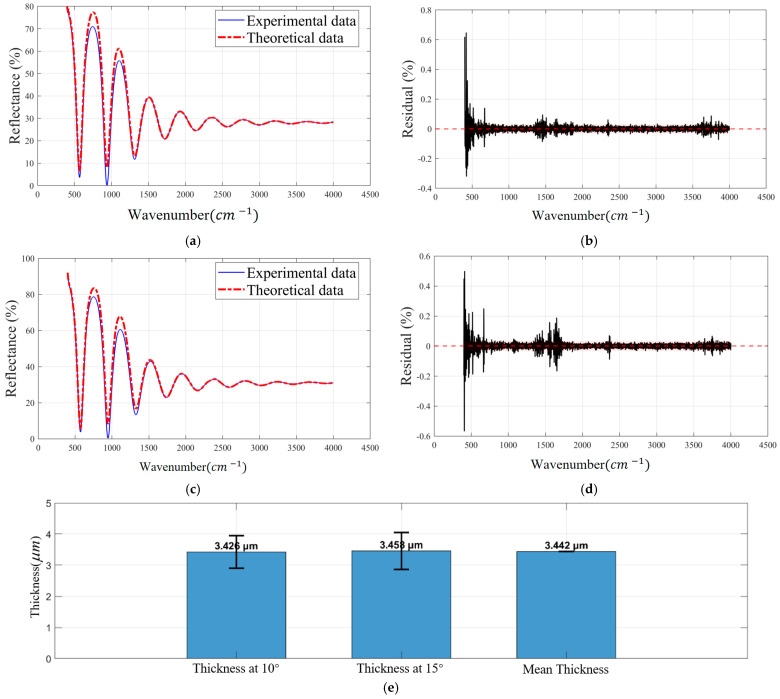
Display of thickness fitting, residuals, and cross-validation: (**a**) reflectance at 10°; (**b**) residual at 10°; (**c**) reflectance at 15°; (**d**) residual at 15°; (**e**) thickness comparison.

Figure 10 demonstrates the performance of the Airy-function-based thickness correction model through fitting results, residual analysis, and cross-validation. The corrected thickness curves exhibit excellent agreement with the experimental data, with a coefficient of determination (R^2^) exceeding 0.99 and a root mean square error (RMSE) below 0.0341%. The residuals are centered around zero with a mean value close to 0 and a standard deviation of 0.0326%, indicating the absence of observable systematic bias. Slightly larger variations in the low-wavenumber region are attributed to the reduced signal-to-noise ratio in this spectral range. In addition, the two independently corrected thickness values, 3.426 μm and 3.458 μm, differ by only 0.032 μm, corresponding to a cross-validation deviation of approximately 0.93%, which further confirms the consistency of the thickness estimates and the robustness of the proposed method. The application of the Airy-function correction reduces the relative error from 1.34% to 0.93% for silicon wafers (Table 6 and Table 7), while for SiC samples, the inter-angle consistency deviation is reduced from 1.14% to 0.08% (Table 3 and Table 4). These results support the effectiveness of the proposed correction approach in this supplementary silicon wafer case and suggest that the multi-beam identification and correction strategy may be transferable to an additional reflectance spectrum scenario.

## 4. Discussion

The results under identical data conditions demonstrate that the proposed method provides substantially improved thickness estimation consistency compared with conventional reflectance-based approaches. As summarized in Table 8, the proposed framework yields thickness values of 8.86 μm and 8.85 μm for the 10° and 15° incident angle datasets, respectively, corresponding to an inter-angle deviation of only 0.08%. In contrast, the FFT-based method produces thickness values of 13.72 μm and 13.85 μm, with a deviation of 0.99%, while the fringe-based method could not provide stable and reproducible thickness values because the spectral extrema were ambiguous. These results indicate that incorporating dispersion compensation and multi-beam interference correction significantly improves the cross-angle consistency of thickness retrieval from infrared reflectance spectra.

To further position the proposed method within the current landscape of SiC thickness metrology, a literature-based comparison with representative techniques is provided in Table 9, including spectroscopic ellipsometry, FTIR reflectometry, optical interferometry, and FFT-based spectral analysis. This comparison shows that spectroscopic ellipsometry remains a widely recognized reference technique because of its high sensitivity and rich data dimensionality, but it requires multi-angle, polarization-resolved measurements and dedicated instrumentation. By contrast, reflectance-based methods are generally more compatible with industrial inspection scenarios, although their performance can be affected by dispersion, peak ambiguity, and multi-beam interference. In this context, the present method is designed as a physics-constrained inversion framework for the practically important scenario of thickness estimation from infrared reflectance spectra. Rather than replacing ellipsometry, it provides a lower-complexity and more deployable solution for applications where standard infrared reflectance spectra are available. Importantly, the conclusions from literature benchmarking (Table 9) and same-dataset evaluation (Table 8) are consistent, both indicating that the proposed method achieves improved robustness and internal consistency within the reflectance-based measurement framework.

In this study, the SiC datasets remain the primary development and validation target, whereas the silicon wafer case should be interpreted only as a supplementary transfer test. Therefore, the present results support broader applicability in principle, but do not yet constitute a complete generalization study across different materials, interface conditions, and measurement environments.

From the algorithmic perspective, the improved performance arises from the coordinated integration of physically constrained modeling and adaptive spectral processing. The Sellmeier dispersion relation accounts for the wavelength dependence of the refractive index, thereby reducing systematic bias associated with fixed-index approximations. Meanwhile, the multi-beam correction strategy mitigates spectral distortions caused by higher-order internal reflections, which are difficult to handle using conventional fringe counting or purely frequency–domain analysis. As a result, the framework avoids over-reliance on local peak identification and instead performs thickness inversion under explicit Fresnel reflection and thin-film interference constraints. This explains why the proposed method remains stable even when conventional fringe-based interpretation becomes unreliable.

The reliability analysis further supports the stability of the proposed framework. As described in Section 3.1.2, Monte Carlo perturbation tests show that the retrieved thickness values remain narrowly distributed under random spectral perturbations, and the fitted spectra exhibit no obvious systematic residual bias. Moreover, the close agreement between the independently retrieved thicknesses at two incident angles provides additional evidence that the fitting process is physically consistent rather than merely overfitting local spectral features. It should nevertheless be emphasized that, because no independent absolute thickness ground truth is available in the current dataset, the present evaluation should be interpreted as a performance-level and consistency-based validation rather than a direct metrological accuracy assessment.

Because no independently calibrated absolute thickness reference is available in the present dataset, the validation in this study should be interpreted as consistency-based rather than as direct metrological accuracy assessment. Specifically, the official reference intervals associated with the dataset, namely 7.5–8.5 μm for the SiC sample and 3–4 μm for the silicon wafer sample, are used only as coarse external plausibility references, rather than as ground truth. Within this constraint, the agreement between the independently measured 10° and 15° spectra is adopted as the primary internal consistency criterion for the SiC case. In addition, the roles of dispersion-aware modeling and multi-beam correction are examined through component-isolation comparisons, and Monte Carlo perturbation tests are used to assess robustness under spectral uncertainty. This layered evidence structure defines the actual validation scope of the present work.

Despite these encouraging results, several limitations remain. First, no independently calibrated absolute thickness reference is available in the present dataset. Therefore, the current results should be interpreted primarily as evidence of improved internal consistency and workflow robustness, rather than as a complete metrological validation of absolute thickness accuracy. Second, the present model assumes an effective homogeneous epitaxial layer and does not explicitly account for surface crystallinity, interface roughness, thickness/refractive index non-uniformity, non-uniform doping distributions, or possible polycrystalline microstructural effects, all of which may influence the reflection and interference behavior in practical samples. These effects may become particularly important when the inferred thickness variation approaches the scale of surface or interface roughness. Future work will therefore focus on extending the model to heterogeneous media, incorporating roughness-related effects, and validating the workflow against independently calibrated thickness measurements such as SEM, ellipsometry, or certified reference samples. In addition, although the silicon wafer case suggests that the multi-beam identification and correction strategy may be transferable beyond the main SiC example, broader validation across different materials and measurement conditions is still needed.

## 5. Conclusions

A physics-constrained inversion framework integrating Savitzky–Golay filtering, Gaussian fitting, dispersion-aware modeling, and adaptive multi-beam correction is developed for SiC epitaxial layer thickness estimation from infrared reflectance spectra. On the present SiC datasets measured at two incident angles, the adaptive multi-beam correction step further improves cross-angle consistency, reducing the inter-angle deviation of the thickness estimates from 1.14% to 0.08%. Monte Carlo perturbation analysis is additionally used to assess the robustness of the inversion results under spectral uncertainty.

The present study is primarily developed and evaluated for the SiC application scenario considered here. Because no independently calibrated absolute thickness reference is available in the current dataset, the present results should be interpreted as evidence of improved internal consistency, plausibility relative to the official reference intervals, and robustness under spectral uncertainty, rather than as a complete validation of absolute metrological accuracy. In particular, the official reference intervals associated with the dataset, namely 7.5–8.5 μm for the SiC sample and 3–4 μm for the silicon wafer sample, are used only as coarse plausibility references rather than as independently calibrated ground truth. A supplementary silicon wafer test suggests that the multi-beam identification and correction strategy may be applicable beyond the main SiC example, but this should not be interpreted as a complete generalization of the present method across different materials and measurement conditions. Broader cross-material validation and independently calibrated reference measurements such as SEM, ellipsometry, or certified reference samples remain necessary in future work.

## Figures and Tables

**Table 1:** Epitaxial layer thickness (nonlinear fitting).

Angle of Incidence	A	B	C	Epitaxial Layer Thickness/μm
10°	7.4847	0.9311	1.7 × 10^−6^	8.8495
15°	7.7072	1.7258	1.1 × 10^−6^	8.9506

**Table 2:** Confidence levels of multiple-beam interference effects.

Angle of Incidence	Confidence Level
10°	72.8%
15°	72.8%

**Table 3:** SiC thickness results after filtering correction.

Angle of Incidence	Monte Carlo/μm	Final Thickness/μm
10°	8.5373 ± 0.1924	8.8617
15°	8.5598 ± 0.2147	8.8546

**Table 4:** Raw silicon carbide thickness results after optimization.

Angle of Incidence	Monte Carlo/μm	Final Thickness/μm
10°	8.9275 ± 0.2091	8.8495
15°	8.9827 ± 0.2868	8.9506

**Table 5:** Comprehensive confidence levels for Attachments S3 and S4.

Data	Comprehensive Confidence
Attachment S3	79.9%
Attachment S4	80.1%

**Table 6:** Calculated thickness results of silicon wafers without the Airy function.

Angle of Incidence	Optimized Thickness
10°	3.828
15°	3.880

**Table 7:** Calculated thickness results of silicon wafers with the Airy function.

Angle of Incidence	Optimized Thickness
10°	3.426
15°	3.458

**Table 8:** Quantitative comparison of thickness estimation methods for the SiC sample under identical conditions.

Method	10° (μm)	15° (μm)	Deviation (%)
Fringe	N/A	N/A	N/A (unstable due to peak ambiguity)
FFT	13.72	13.85	0.99
Ours	8.86	8.85	0.08

**Table 9:** Qualitative comparison of thickness estimation methods for the SiC sample under identical conditions.

Method	Measurement Principle	Data Type	Typical Performance	Reference
Spectroscopic ellipsometry	Polarization + phase (Ψ, Δ)	Multi-angle, polarized	~5% thickness uncertainty	[35]
FTIR reflectometry	Interference spectrum analysis	Reflectance spectrum	Thickness-sensitive for 8–130 nm	[7]
Optical interferometry	Fringe-based thickness extraction	Reflectance/interference signal	~10 nm-level agreement	[36]
FFT-based analysis	Frequency–domain reflectance analysis	Reflectance spectrum	Best suited for films > 1 μm	[37]
Ours	Dispersion compensation + multi-beam correction	Single-angle reflectance	0.08% (inter-angle consistency metric, without absolute ground truth)	-

## Data Availability

The data used in this study, including Attachments S1, S2, S3, and S4, are sourced from the official problem dataset of the 2025 China Undergraduate Mathematical Contest in Modeling (CUMCM 2025). The original dataset (file: CUMCM2025Problems.zip) can be accessed and downloaded from the official CUMCM website: https://www.mcm.edu.cn/index_cn.html (accessed on 2 January 2026).

## Supplementary Materials

The following supporting information can be downloaded at: https://www.mdpi.com/article/10.3390/s26102965/s1.
